# Physical Peculiarity of Two Sites in Human Promoters: Universality and Diverse Usage in Gene Function

**DOI:** 10.3390/ijms25031487

**Published:** 2024-01-25

**Authors:** Kohei Uemura, Takashi Ohyama

**Affiliations:** 1 Major in Integrative Bioscience and Biomedical Engineering, Graduate School of Science and Engineering, Waseda University, 2-2 Wakamatsu-cho, Shinjuku-ku, Tokyo 162-8480, Japan; uemurakohei@fuji.waseda.jp; 2 Department of Biology, Faculty of Education and Integrated Arts and Sciences, Waseda University, 2-2 Wakamatsu-cho, Shinjuku-ku, Tokyo 162-8480, Japan

**Keywords:** core promoter, physical properties of DNA, duplex DNA free energy, GO term, gene organization, immune response

## Abstract

Since the discovery of physical peculiarities around transcription start sites (TSSs) and a site corresponding to the TATA box, research has revealed only the average features of these sites. Unsettled enigmas include the individual genes with these features and whether they relate to gene function. Herein, using 10 physical properties of DNA, including duplex DNA free energy, base stacking energy, protein-induced deformability, and stabilizing energy of Z-DNA, we clarified for the first time that approximately 97% of the promoters of 21,056 human protein-coding genes have distinctive physical properties around the TSS and/or position −27; of these, nearly 65% exhibited such properties at both sites. Furthermore, about 55% of the 21,056 genes had a minimum value of regional duplex DNA free energy within TSS-centered ±300 bp regions. Notably, distinctive physical properties within the promoters and free energies of the surrounding regions separated human protein-coding genes into five groups; each contained specific gene ontology (GO) terms. The group represented by immune response genes differed distinctly from the other four regarding the parameter of the free energies of the surrounding regions. A vital suggestion from this study is that physical-feature-based analyses of genomes may reveal new aspects of the organization and regulation of genes.

## 1. Introduction

All protein-coding genes in eukaryotes are transcribed by RNA polymerase II (Pol II). Although Pol II can synthesize RNA chains, it cannot initiate transcription independently. To execute accurate transcription initiation, Pol II requires assembly of the general transcription factors (GTFs) TFIIA, TFIIB, TFIID, TFIIE, TFIIF, and TFIIH on the promoter DNA [1,2,3]. The resulting large protein complex is called a pre-initiation complex (PIC) [4,5,6,7,8]. The promoters in some PICs harbor core promoter elements (CPEs) of 6–10 bp, which are distinguished by their sequences. CPEs have long been thought to be necessary for accurate transcription initiation [2]. TATA box (Goldberg–Hogness box), initiator (Inr), downstream promoter element (DPE), and motif ten element (MTE) are well-known CPEs [9,10,11,12,13,14].

A longstanding question is how the CPEs in the promoter of a gene to be transcribed initiate PIC formation; these elements are embedded and distributed in much longer genomic DNA comprising more than several G bp in some cases. The TATA box with the consensus sequence TATAWAWR (W; A or T, R; A or G) [15] and Inr with YYANWYY (Y; C or T) [11,12] are both only 2.4 nm in length while the human genome is approximately 1 m. Precisely locating these elements is likened to looking for a needle in a haystack. Target search by transcription factors applies to all *cis*-DNA elements recognized by *trans*-acting factors. A more enigmatic aspect of the search issue is that significant fractions of the Pol II promoters do not contain the TATA box and/or Inr [16,17,18,19]. The same is true for the promoters of long noncoding (lnc)RNA genes and microRNA (miRNA) genes that are also transcribed by Pol II [20]. Furthermore, “core-less” promoters, which do not contain CPEs, are considered to comprise the majority of human Pol II promoters [20,21]. Core-less promoters raise an essential question of how they display their existence to GTFs; this is a fundamental problem, similar to the search for definite “visible” sequences.

Among GTFs, TFIID is the most relevant to the search because it recognizes and binds core promoters irrespective of the presence or absence of the TATA box and nucleates stepwise PIC assembly [22,23]. Proper placement of Pol II relative to the TSS may be governed by TFIID as its primary function via proper positioning of the TATA-binding protein (TBP; a subunit of TFIID) on the core promoter [22]. In TATA-less promoters, accurate loading of TBP is suggested to be ensured by TBP-associated factors (TAFs) 1 and 2 (subunits of TFIID) that bind sites downstream relative to the TSS; to position TBP at a precisely defined upstream site, they collectively act as molecular rulers, making their binding sites a foothold [22]. These studies on PIC assembly [22,23] used artificial promoters, including the super core promoter (SCP) that was designed to increase the affinity of TFIID for DNA and contained the TATA box, Inr, MTE, and DPE [24] or synthetic promoters that carried combinations of the elements, TATA box, Inr, TCT (YYCTTTYY), and DPE. However, the mechanism by which core-less promoters display their presence to TFIID remains unclear.

A hypothesis in this regard is that the physical or structural properties of DNA, rather than the DNA sequence itself, may play an essential role in core promoter recognition by TFIID. Notably, the average flexibility/rigidity properties of human TATA-only promoters, Inr-only promoters, GC-box-only promoters, and core-less promoters, which were calculated using the genes of each group aligned at TSSs, revealed a common distinctive property. Each group had a distinctively flexible and rigid sequence side-by-side at a single definite position, which was the TATA box for TATA-only promoters, Inr for Inr-only promoters, and around the TSS for the other two groups; conversely, no distinctive profile was found in the other parts of the promoter region [17,21]. The distinctive physical properties of the TATA-only and Inr-only promoters originated from the TATA boxes and Inrs, respectively. For the remaining two groups, the same profile emerged around the TSS. However, there was no consensus sequence around this position. Using unsorted human promoters and similar analysis, Gan et al. reported the average profiles of the promoters for 13 structural or physical properties, which revealed the presence of distinctive properties around the TSS and at position −30 relative to the TSS [25]. Biases based on the TATA consensus sequences originating from TATA-containing promoters and Inr consensus sequences originating from Inr-containing promoters should have entered their profiles in definite proportions. Although this issue remained, the singularity of the TATA box region for 3D structural characteristics was demonstrated by Il’icheva et al. [26]. The melting ability and shape parameters, including roll angle, propeller twist, and minor groove width, of DNA are averagely conserved around the TSS or at an upstream site among eukaryotic promoters, irrespective of the presence or absence of distinct CPEs [27]. Furthermore, increasing suggestions regarding the implications of the structural or physical properties of promoter DNA in transcription initiation have led to the development of computer programs that can predict promoters in a genome [25,28,29,30,31,32,33,34,35,36].

Studies on promoter recognition or function have gradually shifted focus from DNA sequences toward the structural or physical properties of DNA. Two specific positions in the Pol II promoters have been identified using average profiles for DNA properties, as described above: short regions around the TSS and a site corresponding to the TATA box [17,20,21,25,26,27,37]. The positions were identified in all cases. However, we still do not know how many promoters have such a distinctive property around the TSS, position ~−27, or both. Another inadequately studied aspect is the relationship between the presence or absence of such profiles and the functional aspects of the genes. This study addresses these issues for the first time.

## 2. Results

### 2.1. Core-Less Promoters Comprise the Majority of Pol II Promoters

**Figure 1:**
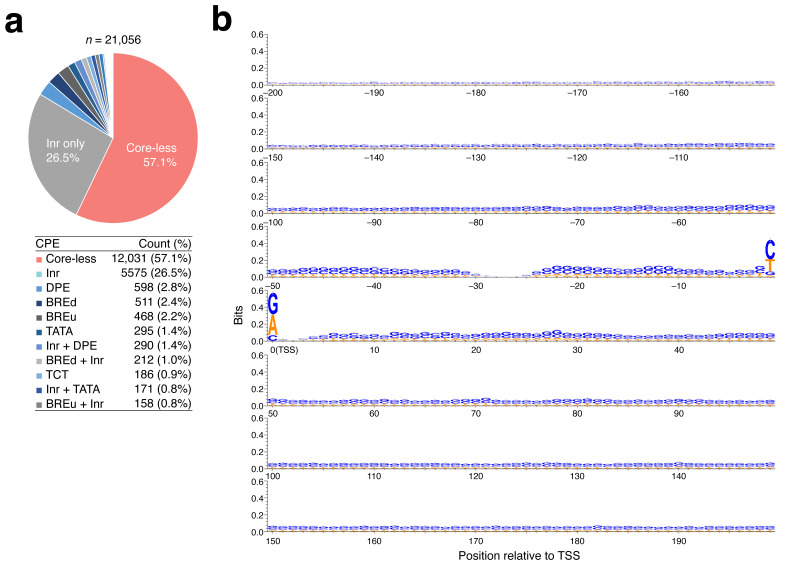
CPE-based assortment of human Pol II promoters. (**a**) Occupancies of the assorted promoters. Those lacking any CPEs are indicated as “core-less”. The top 11 in the population are shown. All data are summarized in Supplementary Table S2. (**b**) Sequence consensus for 12,031 core-less promoters, as examined using the WebLogo3 software [41], at positions −200 to +200 centered at TSS.

The current study focuses only on protein-coding genes and refers to them as Pol II genes hereafter, as per convention, unless this may cause confusion. An early study using 1871 human Pol II promoters suggested that core-less promoters, which lacked canonical promoter elements, such as the TATA box, Inr sequence, and DPE, comprise the majority of human Pol II promoters [21]. Element-based sorting of 21,056 human Pol II promoters was performed to update this knowledge using recent genome databases. We used the coordinates of FANTOM CAT robust transcripts [38] obtained by cap analysis of gene expression (CAGE) [39] to determine promoter positions as a data source. CAGE is the most accurate experimental approach for identifying the 5′ ends of capped RNAs [40]. Based on the differences in CPEs (Supplementary Table S1), we sorted all human Pol II promoters (see Section 4), generating a total of 67 groups (Figure 1a, Supplementary Table S2).

Core-less promoters were the most prevalent among the resulting groups, accounting for approximately 57% of all human Pol II promoters. Using WebLogo3 (ver. 3.7.12) [41], we created a sequence logo for the region from −200 to +200 with respect to the TSSs of the core-less promoters (Figure 1b). Although these promoters showed a slight preference for G or C bases at each nucleotide position and slightly favored C or T (at −1) and A or G (at TSS), no other strong sequence preference was observed. The region from −28 to −26 relative to the TSS did not show any preference. The Inr element, which has a consensus BBCABW (B: C, G, or T) for humans, is located around position −3 to +3 relative to the TSS [18] and is the second most prevalent. However, the percentage of the Inr-containing promoters was 31% (% calculated was based on Supplementary Table S2). The percentage of the well-known TATA box [9], which has a consensus TATAWAWR located around position −31 to −24 relative to the TSS and is conserved from yeast to humans, was calculated to be only 2.5%. As shown in Figure 1a and Supplementary Table S2, except for core-less promoters, each promoter group was found to be a minority.

### 2.2. Average Profiles for Physical Properties of Promoters Highlight Peculiarities of the Regions around the TSS and Position −27

**Figure 2:**
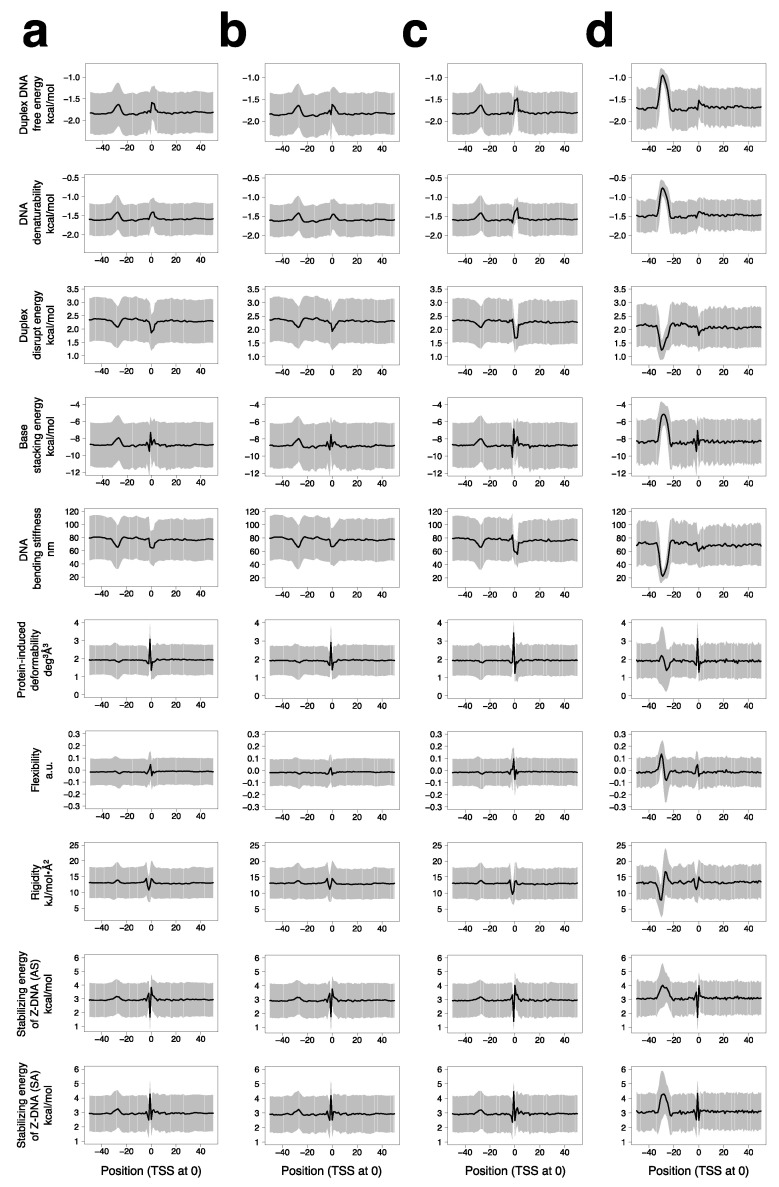
Averaged profiles of human Pol II promoters for 10 DPPs. (**a**) Profiles of all the promoters, (**b**) core-less promoters, (**c**) Inr-containing promoters, and (**d**) TATA-containing promoters; the sample numbers were 21,056, 12,031, 6559, and 533, respectively. Ten DPPs, including duplex DNA free energy [42], DNA denaturability [43], duplex disrupt energy [44], base stacking energy [45], DNA bending stiffness [46], protein-induced deformability [47], flexibility [48], rigidity [49], stabilizing energy of Z-DNA (AS) and stabilizing energy of Z-DNA (SA) [50], and di- to tetra-nucleotide step parameters, were used for calculation. Promoters were aligned with the TSSs assigned at position zero. Values are shown as means ± SD (for numerical values, see Supplementary Tables S3–S6).

Core-less promoters have no consensus sequences. However, Fukue et al. showed that when they were aligned at the TSS, their average flexibility and rigidity profiles demonstrated distinctive features around the TSS [21]. To update the data and prepare the basis for subsequent analyses targeting individual promoters, we prepared the average profiles for 10 DNA physical properties (DPPs) of human Pol II promoter groups, including all 21,056 promoters (Figure 2a), 12,031 core-less promoters (Figure 2b), 6559 Inr-containing promoters (Figure 2c), and 533 TATA-containing promoters (Figure 2d). The calculation involved a sliding window of 10 bp and a moving step of 1 bp (see Section 4). For the groups of the unsorted promoters and core-less promoters, we recently reported their average profiles for duplex DNA free energy, base stacking energy, protein-induced deformability, rigidity, and stabilizing energy of Z-DNA(AS) [20]. The analysis used small fractions of promoter samples (~1/7 of the entire population) obtained by random sampling. Thus, here we performed full-scale analyses again. As shown in Figure 2a,b, there was no difference in the corresponding profiles between the unsorted promoter and core-less promoter groups. Although the population of Inr-containing promoters was not necessarily small, did not generate bias in the DPPs. This can be understood from the profiles shown in Figure 2c. The DPP profiles of the Inr-containing promoters were similar to those of the core-less promoters. TATA-containing promoters showed average DPP profiles (Figure 2d) similar to those shown in Figure 2a–c. However, their distinctiveness in DPPs was highly accentuated at positions approximating −27.

The averaged DPP profiles in Figure 2 are summarized as follows: the TSS region comprising several bps and a small region around −27 were commonly identified as regions with distinctive features; features of the TSS region were remarkably sharp compared to those around −27 in the profiles of protein-induced deformability, the stabilizing energies of Z-DNA (AS and SA), flexibility, rigidity, and stacking energy; the opposite properties coexisted parallelly in the TSS region for these six properties. These characteristics can be used for promoter recognition and functioning [21]. However, a critical aspect to be assessed is the population of promoters that have such profiles on an individual level.

### 2.3. An Energetic Characteristic Lies Near the TSS

**Figure 3:**
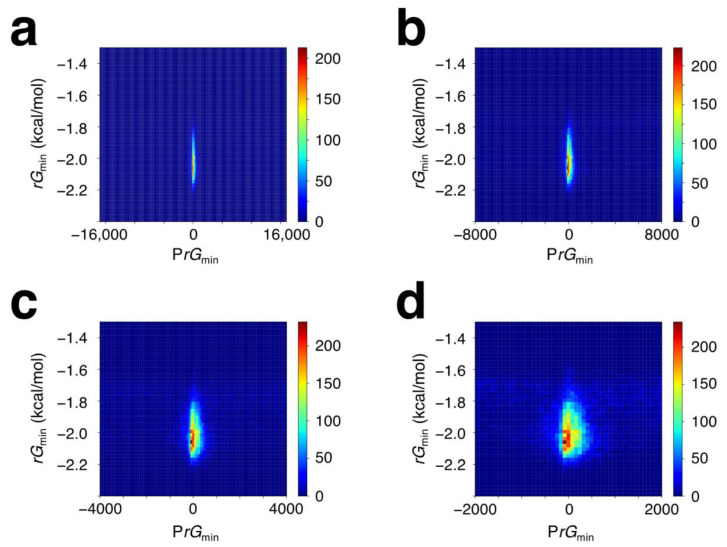
Density 2D plot of *rG*_min_ versus P*rG*_min_. (**a**) Each of the 21,056 human Pol II genes was subjected to the calculation of *rG* over the region from −16 kb to +16 kb, (**b**) from −8 kb to +8 kb, (**c**) from −4 kb to +4 kb, or (**d**) from −2 kb to +2 kb relative to its TSS at 0. The *rG*_min_ value for each gene is plotted against the P*rG*_min_.

We hypothesized that two built-in marks of significantly different sizes might be embedded in the genome to indicate the TSS position of a gene or to act as two functional units that allow transcription initiation; one unit is a comparatively large energetic mark of approximately 150–200 bp (a nucleosomal or promoter DNA size), and the other is a distinctive DPP comprising several bps around the TSS or position −27. Regarding the latter, we hereafter use the singular expression “second built-in mark”. Furthermore, we presumed the free energy of the duplex DNA to be the first marker, as it influences nucleosome occupancy [51]. To substantiate this hypothesis, we first examined whether promoters or promoter-containing wide regions show common energetic features in the human genome. The free energy values for the calculations were based on those reported by Sugimoto et al. [42]. Targeting the TSS-centered 32, 16, 8, and 4 kb sequences, each spectrum of the duplex DNA free energy of a given sequence was calculated with a sliding window of 151 bp and a moving step of 1 bp. The lowest values of the duplex DNA free energy in the target sequence and its position were designated as *rG*_min_ and P*rG*_min_, respectively. These two variables exhibited significant relationships (Figure 3). Despite analyses of considerably wide regions, the data points converged within ±300 bp of the TSS.

### 2.4. Over Half of the TSSs Are Located Close to the PrG_min_ as a Site with the Most Marked Site of Some DPPs

**Figure 4:**
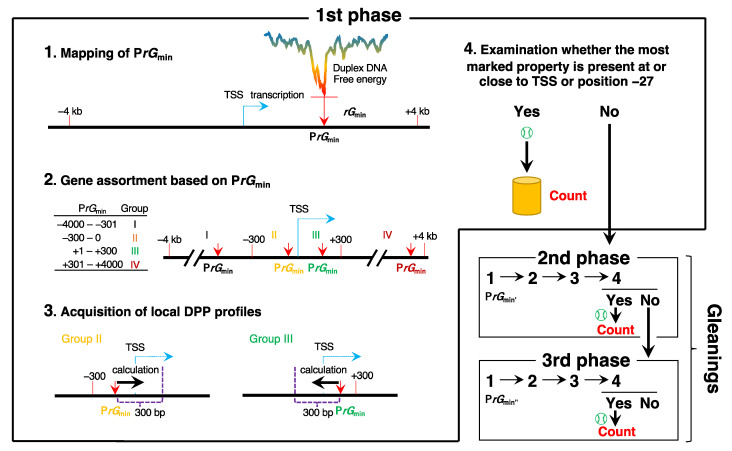
Procedure to substantiate the hypothesis that profiles of *rG* and local DPPs determine the TSS. The procedure comprises three phases. In the first phase, each gene is subjected to the following steps: (**1**) Mapping of P*rG*_min_. An energy scan is performed over a region from −4 kb to +4 kb relative to the TSS (position 0) of a given gene. (**2**) Gene assortment based on P*rG*_min_. Genes are sorted into four groups according to P*rG*_min_: From −4000 to −301, group I; from −300 to 0, group II; from +1 to +300, group III; from +301 to +4000, group IV. (**3**) Acquisition of local DPP profiles. Genes of groups II and III are subjected to the analysis. The calculation starts from the P*rG*_min_ of a given gene and ends at a position 300 bp apart from the P*rG*_min_. The direction of the calculation is opposite between the two groups: group II, toward gene body; group III, toward upstream. (**4**) Screening of genes with the most marked property at or close to TSS or position −27. If some benchmark for any of the ten DPPs (Supplementary Table S7) exists at or close to TSS or position −27 of the gene, it is counted as positive. If not, the gene is subjected to the second phase. Excluding the ±300 bp region centered at P*rG*_min_ and using the new P*rG*_min_ (P*rG*_min′_) in the remaining regions, the same analyses as in the first phase are performed. The third phase targets the negative genes. Excluding ±300 bp region centered at P*rG*_min_ and P*rG*_min′_, the new P*rG*_min_ (P*rG*_min″_) is used in the remaining regions, and the same analysis is performed.

The distinctive DPPs of several bps around the TSS and position −27, as indicated in Figure 2, are the second strongest built-in mark in our hypothesis. However, again, the question of the proportion of individual genes that have such DPPs around the TSS and/or position −27 remained. To determine this, we used the procedure shown in Figure 4. Briefly, in the first phase, all individual 21,056 (all human Pol II genes) or 12,031 (human Pol II genes with a core-less promoter) genes were subjected to four steps of analysis. First, P*rG*_min_ was mapped between −4 kb and +4 kb from the TSS of a given gene (step 1). Then, based on the positional relationship between the TSS and P*rG*_min_, the gene was sorted (step 2). If it belonged to group II (P*rG*_min_; between −300 and 0) or III (P*rG*_min_; between +1 and +300), it was subjected to the next step (step 3), and if not, it was treated as a sample in the second-phase analyses. In step 3, the profiles of 10 DPPs of a 300 bp region illustrated in the figure were obtained. Finally, if the gene had the most significant property at or close to its TSS or position −27 for any of the 10 properties, it was considered a positive sample (step 4). However, if it did not, it was subjected to the second-phase analyses, which employed a new P*rG*_min_, named P*rG*_min′_ (see Section 4). The third-phase analyses were a repetition of the preceding phase analyses; however, they used P*rG*_min″_, a newer P*rG*_min_.

The first-phase analyses clarified that approximately 55% of both gene groups had the two built-in marks as per our hypothesis (Table 1). The “gleanings” (second- and third-phase analyses) gradually elevated the percentages to approximately 75% in both groups, with similar increase rates. Notably, focusing on each set of the corresponding fractionated numbers and percentages, both groups showed similar values (Table 1). Another crucial point to note is that as a second built-in mark, mechanical (***m***) properties (DNA bending stiffness, protein-induced deformability, flexibility, and rigidity; see Materials and Methods), may be employed slightly more than energetic (***e***) and Z-DNA (***z***) properties (***e***: duplex DNA free energy, DNA denaturability, duplex disruption energy, and base stacking energy; ***z***: stabilizing energy of Z-DNA (AS) and stabilizing energy of Z-DNA (SA)). The positions responsible for the positive counts were as follows: for all genes, around TSS: 3320 genes (21.3%), around position −27: 1832 genes (11.7%), and around both positions: 10,452 genes (67.0%); for genes with a core-less promoter, around TSS: 1961 genes (21.7%), around position −27: 1064 genes (11.8%), and around both positions: 6007 genes (66.5%). About two-thirds of the positive genes had distinctive DPPs around the TSS as well as position −27, in which various pairs of DPPs were usually engaged.

### 2.5. Pol II Genes Are Separated into Several Groups Based on Physical Features

**Figure 5:**
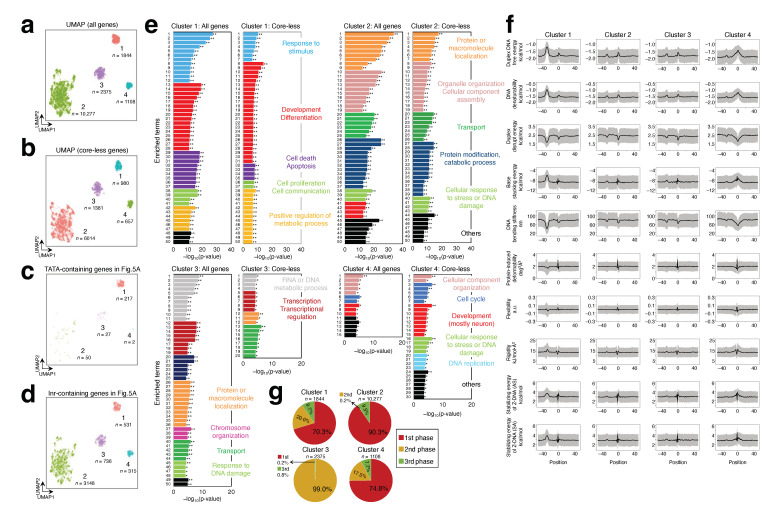
Two-built-in-marks-based clustering of Pol II genes and GO analysis of the resulting clusters. (**a**,**b**) UMAP-DBSCAN clustering of the genes that have the two built-in marks. (**a**) Result for the 15,604 genes screened from unsorted genes. (**b**) Result for the 9032 genes from the genes with a core-less promoter. (**c**) Distribution of the genes with a TATA-containing promoter in (**a**). (**d**) Distribution of the genes with an Inr-containing promoter in (**a**). (**e**) Representative GO BP terms of each cluster in (**a**) (left side) and those in (**b**) (right side). Up to the top 50 enriched terms are shown. *p*-value ≤ 0.05 adjusted by Benjamini–Hochberg procedure was set as the cut-off. The value (*p*-adj) ≤ 0.05 is indicated with one asterisk (*), and less than ≤0.01 is indicated with two asterisks (**). For details of GO enriched terms, see Supplementary Figure S1. (**f**) Averaged DPP profiles of all genes contained in each cluster. Only the data for all genes are shown. For numerical values, see Supplementary Tables S8–S11. (**g**) Proportion of the genes detected in each screening phase in Figure 4. Only the data for all genes are shown.

The analysis of individual promoters strongly suggested that the second built-in mark plays a role in most human Pol II promoters. The next issue to be examined was whether we could classify genes via the detailed features of the two built-in marks. To obtain the answer, a uniform manifold approximation and projection (UMAP) dimension reduction method based on Riemannian geometry and algebraic topology [52] and density-based spatial clustering of applications with noise (DBSCAN) clustering method [53] were applied to 15,604 and 9032 detected genes, respectively (Table 1; the sum of the counts in all phase analyses for all genes and that for genes with a core-less promoter). UMAP is a widely used manifold learning technique. Compared with t-SNE, it is considered to preserve the local and the global data structure more with only a short run time [52]. Thus, it is frequently applied to many genomic studies. DBSCAN is also widely used. This clustering method can group data with similar density into one cluster. Our analysis revealed that the detected genes were separated into four clusters for all genes and genes with a core-less promoter (Figure 5a,b). The distribution of genes with a TATA-containing promoter is shown in Figure 5c; most of these genes were included in cluster 1 of Figure 5a. However, the percentage in cluster 1 was only about 11.8%. In contrast, Figure 5d indicates that the genes with an Inr-containing promoter were almost evenly included in all four clusters.

To characterize each gene cluster in Figure 5a,b, we performed gene ontology (GO) term analysis of the biological process (BP) category (considering up to the top 50 enriched terms; *p*-value cut-off, 0.05). In each set with the same number of clusters, the enriched GO terms of the cluster-forming genes significantly overlapped (Figure 5e and Supplementary Figure S1). Although the same GO terms were sometimes found across clusters, they mostly differed among clusters. Notably, cluster 1 was characterized by such genes as are involved in the development, differentiation, cell death, apoptosis, cell proliferation, and cell communication. For cluster 2, genes involved in protein or macromolecule localization, organization, transport, and modification were conspicuous. Cluster 3 was distinguished from the other clusters by the genes involved in RNA or DNA metabolic processes, transcription, transcriptional regulation, and chromosome organization. For cluster 4, cellular component organization, cell cycle, neuron development, and DNA replication were the common enriched terms between 15,604 and 9032 detected genes (those involved in the cell cycle were found in this cluster separated from 15,604 genes).

Finally, we examined whether each cluster could be explained in terms of the specific characteristics of the built-in marks of cluster-forming genes. As shown in Figure 5f, among clusters 1, 2, and 4, the average DPP profiles of the promoters differed considerably from one another. Conversely, the pie charts, which showed the percentage of the population of the genes detected in each phase of the screening in Figure 4, indicated that they were largely similar (Figure 5g). Therefore, it is likely that clusters 1, 2, and 4 were separated due to differences in promoter DPPs. However, cluster 3 genes were exclusively obtained by the second-phase screening (Figure 5g). Thus, clusters 2 and 3 were presumably separated by the difference in the first built-in mark. This is supported by the data demonstrating that they showed almost identical promoter DPPs (the second built-in mark) to each other (Figure 5f).

**Figure 6:**
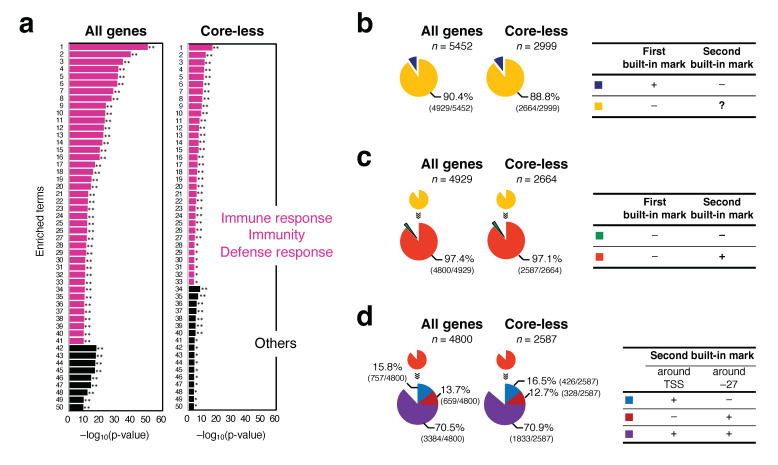
Characterization of undetected genes in the screening. (**a**) GO BP terms of the genes not detected in the screening; left, for 5452 undetected genes among all genes, and right, for 2999 undetected genes among the genes with a core-less promoter. Up to the top 50 enriched terms are shown. A value (*p*-adj) less than ≤0.05 is indicated with one asterisk (*), and less than ≤0.01 is indicated with two asterisks (**). For details of GO enriched terms, see Supplementary Figure S2. (**b**) Percentages of genes whose TSSs were located outside of the P*rG*_min_, P*rG*_min′_, and P*rG*_min″_ territories. (**c**) Percentages of genes whose most distinctive DPPs were located around TSS and/or position −27. (**d**) Occupancies of the TSS and −27 regions in the positive counts.

The screening shown in Figure 4 could not detect 5452 and 2999 genes among all genes and genes with a core-less promoter, respectively. These groups of genes were subjected to GO term analysis in the BP category, which yielded notable findings. They mostly belonged to the category of primary response genes (PRGs) [54], among which those involved in the immune response were significantly enriched (Figure 6a and Supplementary Figure S2). Notably, 90.4% of the 5452 genes (=4929 genes) and 88.8% of the 2999 genes (=2664 genes) had their TSSs outside of the P*rG*_min_, P*rG*_min′_, and P*rG*_min″_ territories (Figure 6b). A majority of the genes in the two groups did not have these regional energy marks. However, 97.4% of the 4929 genes (=4800 genes) and 97.1% of the 2664 genes (=2587 genes) had the most distinctive DPPs around the TSS and/or position −27, i.e., the second built-in mark (Figure 6c). In addition, 70.5% (3384/4800) and 70.9% (1833/2587) were found by the distinctive DPPs around both the TSS and position −27 (Figure 6d). Notably, considering the data on the detected 10,452 (all genes) and 6007 genes (genes with a core-less promoter), among which 67.0% (10,452/15,604) and 66.5% (6007/9032) have distinctive DPPs around both sites, respectively, they indicate that 65.7% ((10,452 + 3384)/21,056) of all human Pol II promoters and 65.2% ((6007 + 1833)/12,031) of core-less promoters have such distinctive properties around both sites; various pairs of DPPs are usually involved in this phenomenon.

## 3. Discussion

In this study, we scrutinized the CPE-based and DPP-based statistics of human Pol II promoters and discuss the implications of our findings. Most of the 21,056 human Pol II promoters have distinctive DPPs around the TSS or position −27, and approximately 65% exhibit such properties around both sites. The same is nearly true for the 12,031 genes with a core-less promoter. These percentages were 1.5-fold higher than for approximately 43% of CPE-containing promoters among the 21,056 genes. Furthermore, the human Pol II genes were separated into five groups based on the detailed features of the two built-in marks.

### 3.1. Percentages of CPE-Containing Promoters Are Low

The percentage of promoters with a focused CPE among the total number of promoters is affected by the database used in the analysis and the various factors used in the screening, such as the employed consensus sequence of the element, inclusion or exclusion of mismatch sequences, and setting of the element position. Early studies generally reported higher percentages of TATA-containing human Pol II promoters compared to recent studies, e.g., ~7% [17], 10.4% [18], 21.8% [55], <~10% [56], 17% [57], and ~10% [18] vs. ~3% [58], 2.1% [27], and 2.5% (this study; Figure 1 and Supplementary Table S2). Vanaja and Yella [27] used the Eukaryotic Promoter Database (EPD) [59]. However, we used FANTOM5 data defined by a robust cut-off [38]. The consensus sequence used for screening was the same in the two groups. Therefore, the slight difference in percentage presumably originates from the difference in data sources. Notably, considering the adequacy of the recent databases, the percentage of TATA-containing promoters among the total number of human Pol II promoters is safely concluded to be about 2–3%.

Percentages of Inr were approximately 45–60% in early studies [18,55,57], while those in recent studies by Vo ngoc et al. and Vanaja and Yella were 40.0% for the focused 7678 promoters [19] and 36.5% for 16,398 promoters [27], respectively; we calculated it to be 31.2% for 21,056 promoters (Supplementary Table S2). The data of Vo ngoc et al. originated from their focused TSSs, which were determined by the 5′-GRO-seq method. Therefore, their percentages cannot be compared with those of Vanaja and Yella or this study. The difference between the values 36.5% and 31.2% was presumed to be primarily due to the difference in the extent of allowance for position deviation, for which Vanaja and Yella allowed a 10 bp deviation from the precisely defined position of −3 to +3 but we allowed a 5 bp deviation.

### 3.2. Almost All Human Pol II Promoters Have the Distinctive DPPs of Several bps around TSS and/or Position −27

Among the core-less promoters, 75.1% had the two built-in marks (Table 1, Supplementary Table S12). Furthermore, among the remaining 24.9% of undetected genes, 86.2% (88.8 × 0.971) had the second built-in mark (Figure 6b,c). Therefore, it follows that 96.6% (75.1 + 24.9 × 0.862) of the human core-less promoters have the second built-in mark (Supplementary Table S12). Similarly, 74.1% of all (unsorted) Pol II promoters had both built-in marks (Table 1, Supplementary Table S12), and 88.0% (90.4 × 0.974) of the remaining 25.9% undetected genes had the second built-in mark (Figure 6b,c), which indicates that 96.9% (74.1 + 25.9 × 0.88) of all human Pol II promoters have the second built-in mark (Supplementary Table S12). Regarding all Pol II promoters, Inr- and TATA-containing promoters intrinsically have this mark (Ref. [17] and Figure 2c,d). Considering the high value of 96.9%, most of the other CPE-containing promoters that occupy 9.2% of all human Pol II promoters (Supplementary Table S2) are thought to have the second built-in mark as well. Collectively, distinctive DPPs of several bps around the TSS and/or position −27 are considered a common signal among the human Pol II promoters, except for approximately 3% of genes.

### 3.3. Which CPEs or DPPs Are Essential for Promoter Function

One of the aims of this study was evaluating whether CPEs or DPPs are essential for promoter function. An early study assessed this using a reporter assay system (in which simian COS-7 cells were transfected with reporter constructs) with its promoters substituted with various synthetic DNA fragments that mimicked the average flexibility profile of core-less promoters [17]. These artificial DNAs showed promoter activity and thus DPPs, not CPEs, were considered essential for promoter function. In silico studies have also been conducted to address this issue. However, since the discovery of the distinctive mechanical properties of Pol II promoters [17], these studies have used average structural or physical properties [25,26,27,37]. Therefore, in silico studies have not yet succeeded in providing clear conclusions. However, the statistical data presented in this study suggest that distinctive DPPs are essential for promoter function.

The question then is, what function do the distinctive DPPs serve? They may function as prerequisites for the conformational changes or strand separation of DNA in PICs. The former is related to the DPPs of several bps around position −27, which corresponds to the site of the TATA box. TBP severely bent the TATA box towards the major groove, producing a wide-open, underwound, shallow minor groove [60,61]. More importantly, TBP similarly bent the TATA box and TATA-less promoters in the PIC [23]. A prescribed change in DNA conformation at a definite position may be achieved by preparing distinctive DPPs. The issue of duplex separation similarly applies to the DPPs around the TSS. The initially melted DNA region (IMR) in the promoter occurs approximately 20–30 bp downstream of the TATA box [62], i.e., around the TSS, forming an open complex from a closed complex. Recently, Dienemann et al. suggested that PIC-induced DNA distortions may prime the IMR for melting and that DNA distortion in the polymerase cleft is a general mechanism contributing to promoter opening [63]. Based on the suggestion and the distinctive DPPs around the TSS in this study, which indicate low duplex stability of the region (Figure 2), we assume that specific DPPs are endowed in this region to facilitate strand separation, as was previously speculated by Il’icheva et al. [26].

Another possibility is that the distinctive DPPs may be related to promoter recognition. TBP, which also binds to TATA-less promoters, may recognize distinctive DPPs around position −27. Similarly, TAFs 1 and 2 may use distinct DPPs around the TSS to locate their binding sites. In this hypothesis, we need not introduce the TAF-1- and TAF-2-based molecular ruler mechanism [22]. Briefly, the distinctive DPPs around the two positions can support the binding of TBP and TAFs 1 and 2 in core-less promoters, aiding most human Pol II promoters in the first step of PIC formation. In light of this hypothesis, the TATA box and Inr sequence seem to have evolved from sequences with distinctive DPPs to meet specialized requirements. However, at present, we cannot refer to what they are.

### 3.4. Human Pol II Genes Can Be Roughly Classified by rG and DPP Profiles

Clustering of the human Pol II genes with the two built-in marks showed they could be grouped into four clusters based on the features of the marks. Importantly, these clusters and a group of undetected genes contained GO terms specific to each (Figure 5e and Figure 6a, and Supplementary Figure S2). Additionally, human Pol II genes can be divided into three classes based on the first built-in mark. The genes detected in the first screening phase formed one class (class 1), accounting for approximately 54–56% (Table 1). Those detected in the second and third phases of screenings (gleanings) formed another class (class 2; ~20%) and undetected genes formed the other class (class 3; ~25–26%). However, the boundaries among these classes were unlikely to be clear. Many genes in clusters 1, 2, and 4 belonged to class 1: cluster 1, 70.3% of genes; cluster 2, 90.3%; cluster 4, 74.8% (Figure 5g). These genes obviously differed in *rG* background from 99.0% of the genes in cluster 3, which belonged to class 2, and all genes in class 3 (a group of undetected genes).

*rG* was calculated using di-nucleotide step values [42] with a sliding window of 151 bp and a moving step of 1 bp (Section 4). Furthermore, P*rG*_min_ corresponded to the center position of the most stable duplex of 151 bp among all segments of the same size in the TSS-centered regions. In class 1 genes, P*rG*_min_ was located within the TSS-centered ±~300 bp region (Figure 3), suggesting that *rG* plays a vital role in the transcription of class 1 genes. The most probable role may be forming a nucleosome-free region (NFR) around the TSS, as low duplex DNA free energy contributes to resistance in the unwinding of the duplex or formation of a negative supercoil compared to duplex DNA with high free energy.

Class 3 genes passed through all filters for P*rG*_min_, P*rG*_min′,_ and P*rG*_min″_. Therefore, *rG* may not have a specific role in transcription initiation in this group, unlike class 1 genes. GO term analysis showed that the genes involved in the immune response and defense response were enriched in this group (Figure 6a and Supplementary Figure S2). These genes are PRGs that are grouped as chromatin remodeler-dependent and -independent [64]; promoters of the latter genes may have unstable nucleosomes, allowing for high constitutive accessibility; however, those of the former may require transcription factors that promote selective nucleosome remodeling [64]. The presence of relatively high *rG* in the core promoter region may lead to the formation of unstable nucleosomes. Furthermore, these chromatin structures may contribute to the rapid and robust responses of the genes to noisy and distinct cellular environments [65].

Class 2 genes were intermediate between classes 1 and 3 regarding their hypothetical *rG* dependency. This class was established by the P*rG*_min′_ and P*rG*_min″_ filters, and the former was an essential factor in the clustering that led to the formation of cluster 3, as indicated in the pie chart (Figure 5g). Among cluster 3 genes, those involved in RNA/DNA metabolic processes, transcription, and transcriptional regulation may be the representatives of this cluster, as elaborated from the enrichment score (Figure 5e).

Regarding the second built-in mark, the average DPP profiles differed significantly among clusters 1, 2 (or 3), and 4 (Figure 5f), indicating that the second built-in mark had a larger contribution to the separation of genes than the first built-in mark in the clustering. This, in turn, strongly suggests that the human genome may divide Pol II genes into functionally different groups by performing fine tuning of DPPs around the TSS and position −27. However, the most important point to remember here is that almost all Pol II genes have a second built-in mark regardless of the class.

## 4. Materials and Methods

### 4.1. Genome Sequence

This study used the human genome assembly hg19, which was obtained from the UCSC Genome Browser [66]. This is because the dataset of TSSs stored in FANTOM CAT [38] was constructed based on the hg19.

### 4.2. Dataset for TSSs

We used a set of high-confidence TSSs defined by the FANTOM5 Consortium [67]. Specifically, the coordinates of the FANTOM CAT robust cut-off [38] were used. They were more strictly defined than those of FANTOM5 and thus form a higher-confidence dataset.

### 4.3. Selection of a Representative TSS among TSS-Seq Reads

The TSS with the highest transcription initiation evidence score (TIEScore), defined by the FANTOM CAT robust cut-off [38], was regarded as the representative TSS of each coding gene. For the analyses, we used the dataset of the representative TSSs thus obtained.

### 4.4. Promoter Classification

The consensus sequences and positions of human Pol II CPEs were obtained from Bucher [15], Vo ngoc et al. [19], Burke and Kadonaga [13], Kutach and Kadonaga [68], Hirose et al. [69], Parry et al. [70], Lagrange et al. [71], Deng and Roberts [72], Tokusumi et al. [73], Anish et al. [74], and Hendrix et al. [75]. Promoter classification was based on the species of CPE(s). In the element search, shifts < 5 bp were allowed for localization. Those lacking CPEs were defined as core-less.

### 4.5. Average DNA Physical Properties (DPPs) of Promoters

Using the di-, tri-, or tetra-nucleotide step values of the DPPs, each sequence was converted into a string of numerical values with a moving step of 1 bp. After collecting data for all promoters (21,056) and core-less promoters (12,031), the mean value and standard deviation at each step were obtained.

### 4.6. Calculation of Duplex DNA Free Energy

The duplex DNA free energy of a given sequence was calculated using di-nucleotide step values reported by Sugimoto et al. [42] with a sliding window of 151 bp and a moving step of 1 bp. The calculation targeted a region of 4, 8, 16, or 32 kb centered at a representative TSS (position 0). The minimum value of the *rG* was denoted as *rG*_min_.

### 4.7. Gene Assortment Based on rG

The position at which *rG* shows the lowest value (P*rG*_min_) was mapped for a region from −4 kb to +4 kb relative to the TSS (position 0) of a given gene. Based on P*rG*_min_, genes were sorted into four groups: −4000 to −301, group I; −300 to 0, group II; +1 to +300, group III; +301 to +4000, group IV.

### 4.8. Acquisition of Local Profiles of DPPs

The group II and III genes described above were subjected to the acquisition of local profiles of DPPs. The calculation started from the P*rG*_min_ of a given gene and ended 300 bp away from the P*rG*_min_. The direction of the calculation was opposite for the two groups; group II was toward the gene body, and group III was toward upstream. The used DPPs were duplex free energy [42], DNA denaturability [43], duplex disruption energy [44], stacking energy [45], DNA bending stiffness [46], protein-induced deformability [47], flexibility [48], rigidity [49], stabilizing energy of Z (AS), and stabilizing energy of Z (SA) [50]. The profile for each property was obtained using a sliding window of 10 bp and a moving step of 1 bp. The di-, tri-, and tetra-nucleotide pair values were averaged within each window (frameshift, 1 bp) and assigned to the center position of the window.

### 4.9. Screening of Genes with Most Marked Property around TSS or Position −27

The local DPPs of groups II and III were obtained as described above. Some averaged profiles of the DPPs had a distinctive peak or trough at a slightly shifted position from the TSS or −27. Therefore, if TSS, position −27, or the positions listed in Supplementary Table S7 of a given gene coincided with a position that had the region-maximum or region-minimum value or the region-maximum or region-minimum change rate of any of the 10 properties described above within an error range of ±10 bp, the gene was judged “positive”.

### 4.10. Gleaning of Genes from Undetected Genes in the First Screening

Excluding the ±300 bp region centered at P*rG*_min_, a new P*rG*_min_ was determined in the remaining 7.4 kb region and named P*rG*_min′_. Then, the same screening described above (1st phase screening) was performed. Furthermore, excluding a ±300 bp region centered at P*rG*_min_ and that at P*rG*_min′_, a new P*rG*_min_ was determined in the remaining 6.8 kb region, named P*rG*_min″_, and the same screening as above was repeated.

### 4.11. DPP Scans of Promoters Whose TSSs Were Outside of PrG_min_, PrG_min′_, and PrG_min″_ Territories

Using the genes whose TSSs were outside the territories of P*rG*_min_, P*rG*_min′,_ and P*rG*_min″_ in the screenings described above, their DPPs of 301 bp were examined as described above, with their TSS aligned at the center.

### 4.12. Dimensionality Reduction Algorithm

Uniform manifold approximation and projection (UMAP) [52] was performed using the R package umap (ver. 0.2.10.0) with default parameters. UMAP was applied to the 15,604 genes screened from unsorted genes with input hyperparameters (n_neighbors = 15, min_dist = 0.1) and the 9032 genes screened from the genes with a core-less promoter with input hyperparameters (n_neighbors = 16, min_dist = 0.1). The UMAP data consisted of binary features *rG*_min_, *rG*_min′_, and *rG*_min″_, which denote the hierarchical order of energy in the screening and those of DPPs at or close to the TSS, position –27, or shifted positions, as shown in Supplementary Table S7. Regarding the DPPs, when a given value recorded the region-maximum or region-minimum value or region-maximum or region-minimum change rate, “1” was given; if not, “0” was given.

### 4.13. Clustering and Visualization

The density-based spatial clustering of applications with noise (DBSCAN) [53] clustering method was performed on the UMAP embedding with input parameters epsilon = 1.5 and minimum point (minPts) = 5.

### 4.14. GO Analysis

GO term enrichment analysis was performed using the Database for Annotation, Visualization, and Integrated Discovery (DAVID Knowledgebase v2023q1 [76,77,78]). The enriched GO terms with one-sided *p*-value ≤ 0.05 were considered significant; up to the 50 top enriched terms were listed. The *p*-values were adjusted using the Benjamini–Hochberg method. For GO term enrichment analyses of biological processes (BPs), the GO fat category (GOTERM_BP_FAT) was used for GO term enrichment analysis of BPs.

## 5. Conclusions

Nearly all human Pol II promoters have distinct DPPs around the TSS and/or position −27. Conversely, the proportion of CPE-containing promoters among the total number of human Pol II promoters of 21,056 is ~43%. Therefore, it is strongly suggested that the former (the second built-in mark) plays much more significant universal roles in transcription than CPEs; it may have an evolutionarily older origin than CPEs. The same seems true for transcription of lncRNA genes [20,79], miRNA genes [20], and even tRNA genes [20]. Notably, the human protein-coding genes were divided into five groups of different representative GO terms based on the detailed features of P*rG*_min_ and distinctive DPPs. Although we used the FANTOM CAT database that used the hg19 as a reference sequence [38] in the analyses, the DPP-based analyses using updated versions of this database with the latest version of the human genome assembly would lead to elaboration of the findings reported here. In conclusion, a vital suggestion from this study is that physical-feature-based analyses of genomes may reveal new aspects of the organization and regulation of genes.

## Figures and Tables

**Table 1:** The number and percentage of the genes that have the hypothetical two built-in marks in the promoter.

	All Genes (*n* = 21,056)								Genes with a Core-Less Promoter (*n* = 12,031)							
Detected by	1st Phase		2nd Phase		3rd Phase		All Phases		1st Phase		2nd Phase		3rd Phase		All Phases	
	Count	%	Count	%	Count	%	Count	%	Count	%	Count	%	Count	%	Count	%
***e*** properties ^1^ only	449	2.1	115	0.5	47	0.2	611	2.9	290	2.4	71	0.6	30	0.2	391	3.2
***m*** properties ^2^ only	1088	5.2	286	1.4	120	0.6	1494	7.1	626	5.2	169	1.4	57	0.5	852	7.1
***z*** properties ^3^ only	277	1.3	62	0.3	22	0.1	361	1.7	170	1.4	41	0.3	13	0.1	224	1.9
Both ***e*** and ***m*** properties	1642	7.8	447	2.1	204	1.0	2293	10.9	972	8.1	256	2.1	109	0.9	1337	11.1
Both ***e*** and ***z*** properties	876	4.2	195	0.9	66	0.3	1137	5.4	520	4.3	130	1.1	36	0.3	686	5.7
Both ***m*** and ***z*** properties	812	3.9	238	1.1	97	0.5	1147	5.4	461	3.8	129	1.1	48	0.4	638	5.3
All (***e***, ***m***, and ***z***) properties	6268	29.8	1588	7.5	705	3.3	8561	40.7	3646	30.3	898	7.5	360	3.0	4904	40.8
The sum	11,412	54.2	2931	13.9	1261	6.0	15,604	74.1	6685	55.6	1694	14.1	653	5.4	9032	75.1

^1^ Duplex free energy, DNA denaturability, duplex disrupt energy, and base stacking energy. ^2^ DNA bending stiffness, protein-induced deformability, flexibility, and rigidity. ^3^ Stabilizing energy of Z-DNA (AS) and stabilizing energy of Z-DNA (SA).

## Data Availability

Source data for figures are provided with the paper. The data that support the findings of this study are available from the corresponding author upon reasonable request.

## Supplementary Materials

The following supporting information can be downloaded at: https://www.mdpi.com/article/10.3390/ijms25031487/s1.

## Abbreviations

BP: biological process, CAGE: cap analysis of gene expression, CPE: core promoter element, DBSCAN: density-based spatial clustering of applications with noise, DPE: downstream promoter element, DPP: DNA physical property, GO: gene ontology, GTF: general transcription factor, IMR: initially melted DNA region, Inr: initiator, MTE: motif ten element, NFR: nucleosome-free region, PIC: pre-initiation complex, P*rG*_min_: position of *rG*_min_, *rG*: regional duplex DNA free energy, *rG*_min_: the lowest value of *rG*, SCP: super core promoter, TAF: TBP-associated factor, TSS: transcription start site, UMAP: uniform manifold approximation and projection, Z-DNA (AS): Z-DNA (anti-syn), Z-DNA (SA): Z-DNA (syn-anti).
