# Supplementary material for: Physical Peculiarity of Two Sites in Human Promoters: Universality and Diverse Usage in Gene Function
Source: Int J Mol Sci. 2024 Jan 25;25(3):1487. doi: 10.3390/ijms25031487 (PMC10855393; doi:10.3390/ijms25031487)
Supplement: Supplementary file 1 [file ijms-25-01487-s001.zip › ijms-2789642-supplementary.pdf]

Supplementary Tables and Figures

Physical Peculiarity of Two Sites in Human Promoters: Universality and Diverse Usage in Gene Function

Kohei Uemura[1] and **Takashi Ohyama** [1,2,*]

[1] Major in Integrative Bioscience and Biomedical Engineering, Graduate School of Science and Engineering, Waseda University, 2-2Wakamatsu-cho, Shinjuku-ku, Tokyo 162-8480, Japan; uemurakohei@fuji.waseda.jp

[2] Department of Biology, Faculty of Education and Integrated Arts and Sciences, Waseda University, 2-2 Wakamatsu-cho, Shinjuku-ku, Tokyo 162-8480, Japan

* Correspondence: ohyama@waseda.jp

Contents

Supplementary Table S1. Known human CPEs for transcription by Pol II.

Supplementary Table S2. Ratios of CPE(s)-containing human Pol II gene promoters.

Supplementary Table S3. Numerical values for DPPs at each position of the aligned all human Pol II gene promoters.

Supplementary Table S4. Numerical values for DPPs at each position of the aligned core-less promoters.

Supplementary Table S5. Numerical values for DPPs at each position of the aligned Inr-containing promoters.

Supplementary Table S6. Numerical values for DPPs at each position of the aligned TATA-containing promoters.

Supplementary Table S7. Canonical position(s) for the occurrence of distinctive DPPs.

Supplementary Table S8. Numerical values for DPPs at each position of the aligned promoters of the cluster 1 genes in Fig. 5a.

Supplementary Table S9. Numerical values for DPPs at each position of the aligned promoters of the cluster 2 genes in Fig. 5a.

Supplementary Table S10. Numerical values for DPPs at each position of the aligned promoters of the cluster 3 genes in Fig. 5a.

Supplementary Table S11. Numerical values for DPPs at each position of the aligned promoters of the cluster 4 genes in Fig. 5a.

Supplementary Table S12. Proportions of two built-in marks.

Supplementary Figure S1. Non-abbreviated version of Fig. 5e.

Supplementary Figure S2. Non-abbreviated version of Fig. 6a.

Supplementary Table S1. Known human CPEs for transcription by Pol II.

CPE[1]	Consensus sequence[2]	Position[3]	Reference
TATA box	TATAWAWR	–31 - –24	Goldberg, M. L., Thesis, Stanford University, Stanford, CA, USA, 1979
			Bucher, P., *J. Mol. Biol.* **1990,** *212*, 563–578
Inr	BBCABW	–3 - +3	Javahery, R.; et al., *Mol. Cell Biol.* **1994**, *14*, 116–127
			Smale, S. T.; et al., *Cold Spring Harb. Symp. Quant. Biol.* **1998***, 63,* 21–31
TCT	YYCTTTYY	−2 - +6	Parry, T. J.; et al., *Genes Dev.* **2010**, *24*, 2013–2018
DPE (RGWCGTG)	RGWCGTG	+28 - +34	Burke, T. W.; Kadonaga, J. T., *Genes Dev.* **1997***, 11,* 3020-3031
DPE (RGWYVT)	RGWYVT	+28 - +33	Burke, T. W.; Kadonaga, J. T., Genes Dev. **1997**, 11, 3020-3031
BREu	SSRCGCC	–38 - –32	Lagrange, T.; et al., *Genes Dev*. **1998**, *12*, 34–44
BREd	RTDKKKK	–23 - –17	Deng, W.; Roberts, S.G.E.; *Genes Dev*. **2005**, *19*, 2418–2423
DRE	WATCGATW	–100 - –1	Hirose, F.; et al., *J. Biol. Chem*. **1993**, *268*, 2092–2099
XCPE1	DSGYGGRASM	–8 - +2	Tokusumi, Y.; et al., *Mol. Cell. Biol*. **2007**, *27*, 1844–1858
XCPE2	VCYCRTTRCMY	–9 - + 2	Anish, R.; et al., *PLoS ONE* **2009**, *4*, e5103
Pause button	KCGRWCG	+25 - +35	Hendrix, D.A.; et al., *Proc. Natl Acad. Sci. USA* **2008**, *105*, 7762–7767
DCEI	CTTC	+6 - +11	Lewis, B. A., et al., *Proc. Natl Acad. Sci. USA* **2000**, *97*, 7172–7177
DCEII	CTGT	+16 - +21	Lewis, B. A., et al., *Proc. Natl Acad. Sci. USA* **2000**, *97*, 7172–7177
DCEIII	AGC	+30 - +34	Lewis, B. A., et al., *Proc. Natl Acad. Sci. USA* **2000**, *97*, 7172–7177

[1]Core promoter element, [2]B = C or G or T; D = A or G or T; K = G or T; M = A or C; R = A or G; S = C or G;V = A or C or G; W = A or T; Y= C or T.

[3]Relative to the TSS.

Supplementary Table S2. Ratios of CPE(s)-containing human Pol II gene promoters.

CPE(s)[1]	Count	Ratio (%)
Core-less	12,031	57.1
Inr	5,575	26.5
DPE	598	2.8
BREd	511	2.4
BREu	468	2.2
TATA	295	1.4
Inr + DPE	290	1.4
BREd + Inr	212	1.0
TCT	186	0.9
Inr + TATA	171	0.8
BREu + Inr	158	0.8
Pause button	151	0.7
XCPE1	58	0.3
Inr + Pause button	41	0.2
TATA + DPE	31	0.1
Inr + TCT	29	0.1
BREd + DPE	28	0.1
DCE	19	0.1
Inr + DCE	17	0.1
TCT + DPE	14	0.1
BREd + TATA	13	0.1
BREd + Inr + DPE	12	0.1
BREu + Pause button	11	0.1
BREd + BREu	9	0.0
BREd + TCT	9	0.0
BREu + DPE	9	0.0
Pause button + DPE	8	0.0
Inr + TATA + DPE	7	0.0
Inr + XCPE1	7	0.0
DRE	6	0.0
BREd + XCPE1	5	0.0
BREu + XCPE1	5	0.0
Inr + Pause button + DPE	5	0.0
Inr + XCPE2	5	0.0
BREd + Inr + TATA	4	0.0
BREu + Inr + DPE	4	0.0
BREu + Inr + Pause button + DPE	4	0.0
BREu + TCT	4	0.0
BREd + BREu + Inr	3	0.0
BREd + Inr + TCT	3	0.0

BREd + Pause button	3	0.0
Inr + TCT + DPE	3	0.0
TATA + TCT	3	0.0
TATA + TCT + DPE	3	0.0
BREd + DCE	2	0.0
BREu + Inr + Pause button	2	0.0
BREu + TCT + DPE	2	0.0
Pause button + XCPE1	2	0.0
XCPE2	2	0.0
BREd + BREu + DPE	1	0.0
BREd + Inr + Pause button	1	0.0
BREd + TATA + XCPE1	1	0.0
BREd + XCPE1 + DPE	1	0.0
BREu + Inr + XCPE1	1	0.0
BREu + Pause button + DPE	1	0.0
BREu + Pause button + XCPE1	1	0.0
BREu + TATA	1	0.0
BREu + XCPE2	1	0.0
DCE + DPE	1	0.0
DRE + Inr + TCT	1	0.0
DRE + TATA	1	0.0
Inr + DCE + DPE	1	0.0
Inr + Pause button + TATA + DPE	1	0.0
Inr + TATA + DCE	1	0.0
Inr + XCPE1 + DPE	1	0.0
Pause button + TATA	1	0.0
XCPE1 + DPE	1	0.0
TATA-containing promoters	533	2.5
Inr-containing promoters	6,559	31.2
Other CPE-containing promoters	1,933	9.2

[1]Core promoter element(s).

Supplementary Table S3. Numerical values for DPPs at each position of the aligned all human Pol II gene promoters.

<separate .xlsx file>

Supplementary Table S4. Numerical values for DPPs at each position of the aligned core-less promoters.

<separate .xlsx file>

Supplementary Table S5. Numerical values for DPPs at each position of the aligned Inr-containing promoters.

<separate .xlsx file>

Supplementary Table S6. Numerical values for DPPs at each position of the aligned TATA-containing promoters.

<separate .xlsx file>

Supplementary Table S7. Canonical position(s) for the occurrence of distinctive DPPs.

DPPs	Group	Maximum value	Minimum value	Maximum rate of change	Minimum rate of change
DNA denaturability	***e***	+1 or −27	—	+2 or −26	−2 or −31
Duplex disrupt energy	***e***	—	0 or −27	−1 or −31	+2 or −26
Duplex DNA free energy	***e***	0 or −27	—	+2 or −26	−1 or −31
Base stacking energy	***e***	−1 or −27	−2	−1 or −25	−2 or −32
DNA bending stiffness	***m***	—	+1 or −27	−2 or −32	+2 or −26
Protein-induced deformability	***m***	−1	0 or −26	−1 or −29	−2 or −26
Flexibility	***m***	−1 or −30	0, −4 or −26	−1 or −28	0 or −25
Rigidity	***m***	−4, 0 or −27	−2	−4 or −26	−1 or −29
Stabilizing energy of Z DNA (AS)	***z***	0, −2 or −28	−1	−2 or −26	−1 or −31
Stabilizing energy of Z DNA (SA)	***z***	−1 or −27	−2 or 0	−1 or −25	−2 or −32

Supplementary Table S8. Numerical values for DPPs at each position of the aligned promoters of the cluster 1 genes in Fig. 5a.

<separate .xlsx file>

Supplementary Table S9. Numerical values for DPPs at each position of the aligned promoters of the cluster 2 genes in Fig. 5a.

<separate .xlsx file>

Supplementary Table S10. Numerical values for DPPs at each position of the aligned promoters of the cluster 3 genes in Fig. 5a.

<separate .xlsx file>

Supplementary Table S11. Numerical values for DPPs at each position of the aligned promoters of the cluster 4 genes in Fig. 5a.

<separate .xlsx file>

Supplementary Table S12. Proportions of two built-in marks.

The 1st mark	The 2nd mark	All genes		Genes with a core-less promoter	
		Count	Ratio (%)	Count	Ratio (%)
+	+	15,604	74.1	9,032	75.1
+	–	523	2.5	335	2.8
+ or –	+	20,404	96.9	11,619	96.6
–	+	4,800	22.8	2,587	21.5
–	–	129	0.6	77	0.6
+	+ or –	16,127	76.6	9,109	75.7

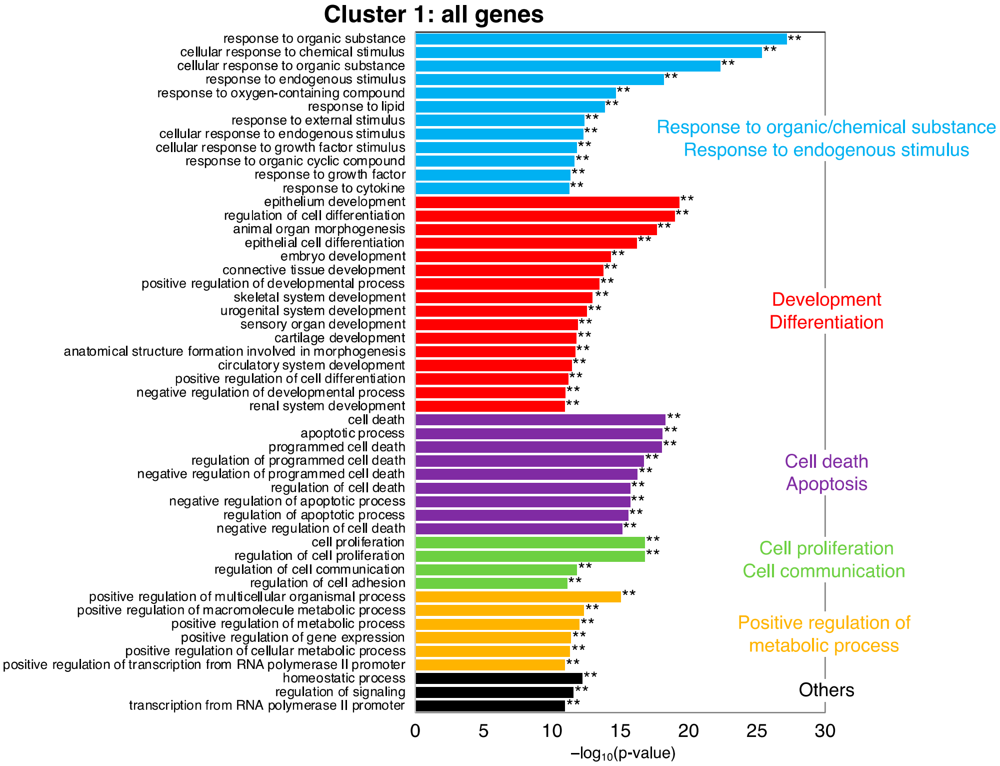
Cluster 1: all genes
response to organic substance
cellular response to chemical stimulus
cellular response to organic substance
response to endogenous stimulus
response to oxygen-containing compound
response to lipid
response to external stimulus
cellular response to endogenous stimulus
cellular response to growth factor stimulus
response to organic cyclic compound
response to growth factor
response to cytokine
epithelium development
regulation of cell differentiation
animal organ morphogenesis
epithelial cell differentiation
embryo development
connective tissue development
positive regulation of developmental process
skeletal system development
urogenital system development
sensory organ development
cartilage development
anatomical structure formation involved in morphogenesis
circulatory system development
positive regulation of cell differentiation
negative regulation of developmental process
renal system development
cell death
apoptotic process
programmed cell death
regulation of programmed cell death
negative regulation of programmed cell death
regulation of cell death
negative regulation of apoptotic process
regulation of apoptotic process
negative regulation of cell death
cell proliferation
regulation of cell proliferation
regulation of cell communication
regulation of cell adhesion
positive regulation of multicellular organismal process
positive regulation of macromolecule metabolic process
positive regulation of metabolic process
positive regulation of gene expression
positive regulation of cellular metabolic process
positive regulation of transcription from RNA polymerase II promoter
homeostatic process
regulation of signaling
transcription from RNA polymerase II promoter
Response to organic/chemical substance
Response to endogenous stimulus
Development
Differentiation
Cell death
Apoptosis
Cell proliferation
Cell communication
Positive regulation of
metabolic process
Others
0
5
10
15
20
25
30
$-\log_{10}$(p-value)

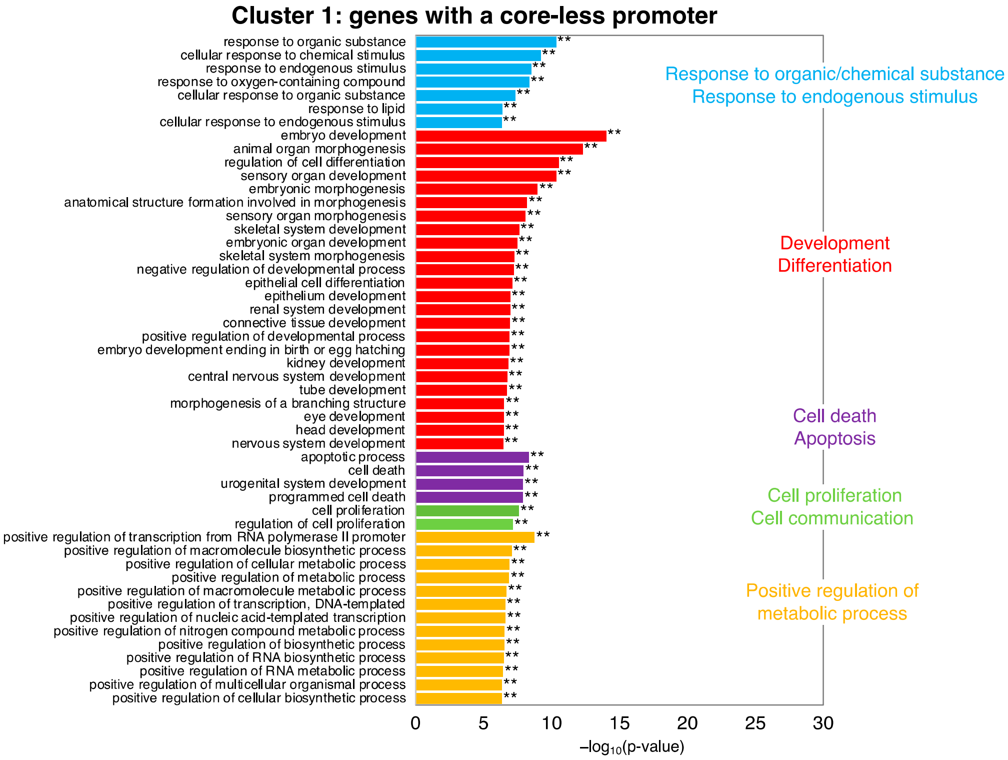
Cluster 1: genes with a core-less promoter
response to organic substance
cellular response to chemical stimulus
response to endogenous stimulus
response to oxygen-containing compound
cellular response to organic substance
response to lipid
cellular response to endogenous stimulus
embryo development
animal organ morphogenesis
regulation of cell differentiation
sensory organ development
embryonic morphogenesis
anatomical structure formation involved in morphogenesis
sensory organ morphogenesis
skeletal system development
embryonic organ development
skeletal system morphogenesis
negative regulation of developmental process
epithelial cell differentiation
epithelium development
renal system development
connective tissue development
positive regulation of developmental process
embryo development ending in birth or egg hatching
kidney development
central nervous system development
tube development
morphogenesis of a branching structure
eye development
head development
nervous system development
apoptotic process
cell death
urogenital system development
programmed cell death
cell proliferation
regulation of cell proliferation
positive regulation of transcription from RNA polymerase II promoter
positive regulation of macromolecule biosynthetic process
positive regulation of cellular metabolic process
positive regulation of metabolic process
positive regulation of macromolecule metabolic process
positive regulation of transcription, DNA-templated
positive regulation of nucleic acid-templated transcription
positive regulation of nitrogen compound metabolic process
positive regulation of biosynthetic process
positive regulation of RNA biosynthetic process
positive regulation of RNA metabolic process
positive regulation of multicellular organismal process
positive regulation of cellular biosynthetic process
Response to organic/chemical substance
Response to endogenous stimulus
Development
Differentiation
Cell death
Apoptosis
Cell proliferation
Cell communication
Positive regulation of
metabolic process
0
5
10
15
20
25
30
$-\log_{10}$(p-value)

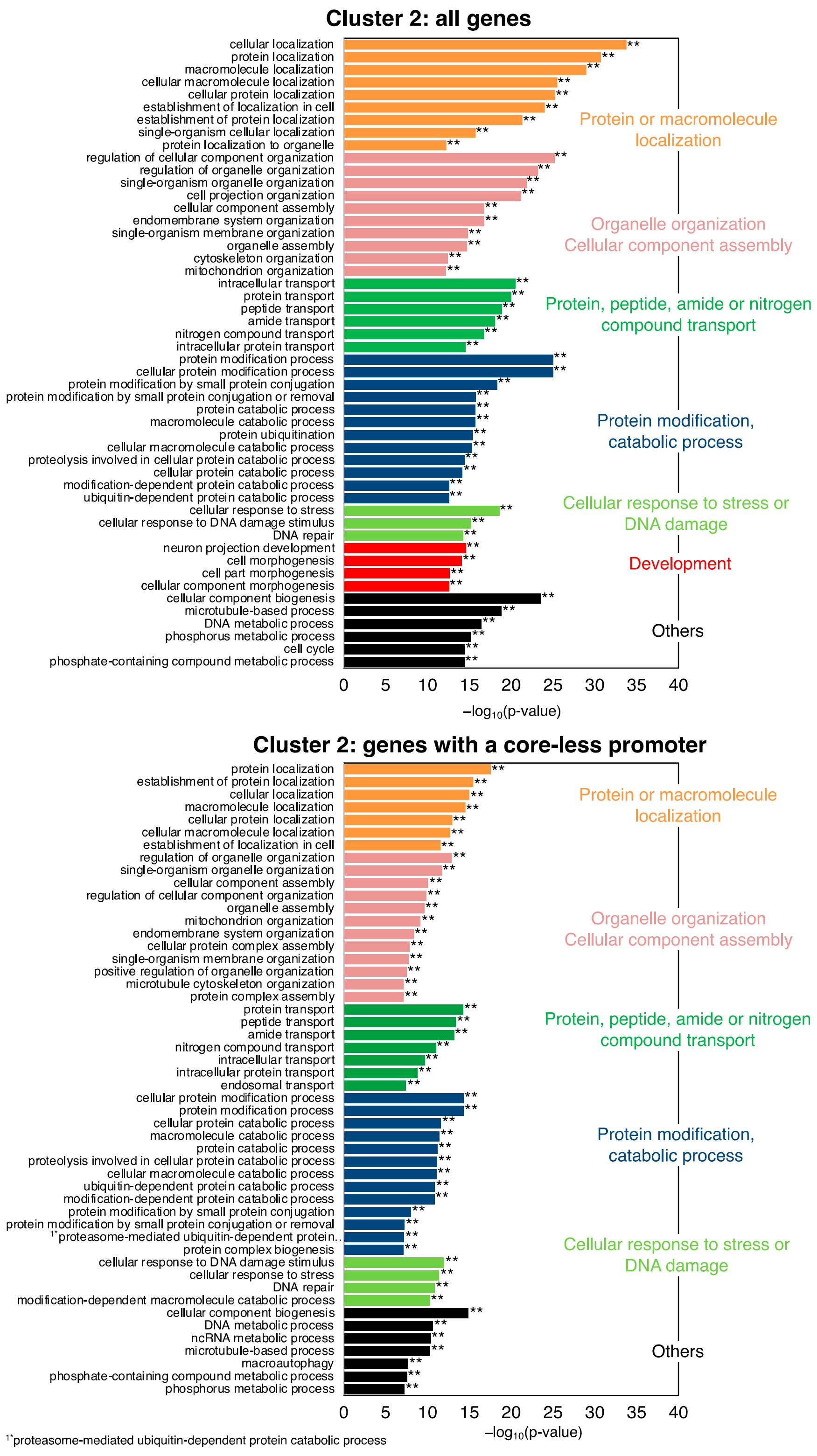
Cluster 2: all genes
cellular localization
protein localization
macromolecule localization
cellular macromolecule localization
cellular protein localization
establishment of localization in cell
establishment of protein localization
single-organism cellular localization
protein localization to organelle
regulation of cellular component organization
regulation of organelle organization
single-organism organelle organization
cell projection organization
cellular component assembly
endomembrane system organization
single-organism membrane organization
organelle assembly
cytoskeleton organization
mitochondrion organization
intracellular transport
protein transport
peptide transport
amide transport
nitrogen compound transport
intracellular protein transport
protein modification process
cellular protein modification process
protein modification by small protein conjugation
protein modification by small protein conjugation or removal
protein catabolic process
macromolecule catabolic process
protein ubiquitination
cellular macromolecule catabolic process
proteolysis involved in cellular protein catabolic process
cellular protein catabolic process
modification-dependent protein catabolic process
ubiquitin-dependent protein catabolic process
cellular response to stress
cellular response to DNA damage stimulus
DNA repair
neuron projection development
cell morphogenesis
cell part morphogenesis
cellular component morphogenesis
cellular component biogenesis
microtubule-based process
DNA metabolic process
phosphorus metabolic process
cell cycle
phosphate-containing compound metabolic process
Protein or macromolecule localization
Organelle organization
Cellular component assembly
Protein, peptide, amide or nitrogen compound transport
Protein modification, catabolic process
Cellular response to stress or DNA damage
Development
Others
0 5 10 15 20 25 30 35 40
$-\log_{10}$(p-value)
Cluster 2: genes with a core-less promoter
protein localization
establishment of protein localization
cellular localization
macromolecule localization
cellular protein localization
cellular macromolecule localization
establishment of localization in cell
regulation of organelle organization
single-organism organelle organization
cellular component assembly
regulation of cellular component organization
organelle assembly
mitochondrion organization
endomembrane system organization
cellular protein complex assembly
single-organism membrane organization
positive regulation of organelle organization
microtubule cytoskeleton organization
protein complex assembly
protein transport
peptide transport
amide transport
nitrogen compound transport
intracellular transport
intracellular protein transport
endosomal transport
cellular protein modification process
protein modification process
cellular protein catabolic process
macromolecule catabolic process
protein catabolic process
proteolysis involved in cellular protein catabolic process
cellular macromolecule catabolic process
ubiquitin-dependent protein catabolic process
modification-dependent protein catabolic process
protein modification by small protein conjugation
protein modification by small protein conjugation or removal
1*proteasome-mediated ubiquitin-dependent protein...
protein complex biogenesis
cellular response to DNA damage stimulus
cellular response to stress
DNA repair
modification-dependent macromolecule catabolic process
cellular component biogenesis
DNA metabolic process
ncRNA metabolic process
microtubule-based process
macroautophagy
phosphate-containing compound metabolic process
phosphorus metabolic process
Protein or macromolecule localization
Organelle organization
Cellular component assembly
Protein, peptide, amide or nitrogen compound transport
Protein modification, catabolic process
Cellular response to stress or DNA damage
Others
0 5 10 15 20 25 30 35 40
$-\log_{10}$(p-value)
1*proteasome-mediated ubiquitin-dependent protein catabolic process

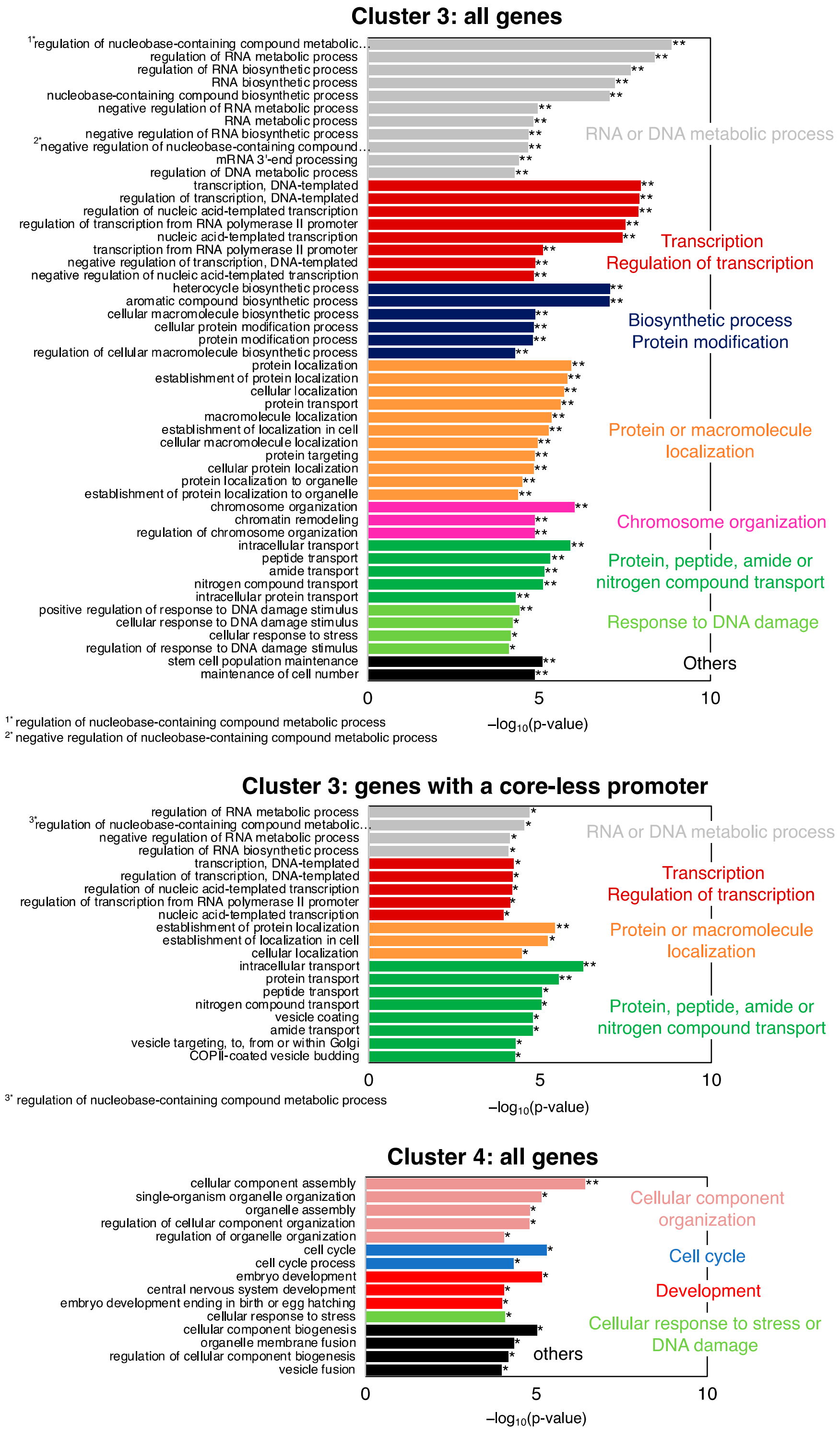
Cluster 3: all genes
1*regulation of nucleobase-containing compound metabolic...
regulation of RNA metabolic process
regulation of RNA biosynthetic process
RNA biosynthetic process
nucleobase-containing compound biosynthetic process
negative regulation of RNA metabolic process
RNA metabolic process
negative regulation of RNA biosynthetic process
2*negative regulation of nucleobase-containing compound...
mRNA 3'-end processing
regulation of DNA metabolic process
transcription, DNA-templated
regulation of transcription, DNA-templated
regulation of nucleic acid-templated transcription
regulation of transcription from RNA polymerase II promoter
nucleic acid-templated transcription
transcription from RNA polymerase II promoter
negative regulation of transcription, DNA-templated
negative regulation of nucleic acid-templated transcription
heterocycle biosynthetic process
aromatic compound biosynthetic process
cellular macromolecule biosynthetic process
cellular protein modification process
protein modification process
regulation of cellular macromolecule biosynthetic process
protein localization
establishment of protein localization
cellular localization
protein transport
macromolecule localization
establishment of localization in cell
cellular macromolecule localization
protein targeting
cellular protein localization
protein localization to organelle
establishment of protein localization to organelle
chromosome organization
chromatin remodeling
regulation of chromosome organization
intracellular transport
peptide transport
amide transport
nitrogen compound transport
intracellular protein transport
positive regulation of response to DNA damage stimulus
cellular response to DNA damage stimulus
cellular response to stress
regulation of response to DNA damage stimulus
stem cell population maintenance
maintenance of cell number
RNA or DNA metabolic process
Transcription
Regulation of transcription
Biosynthetic process
Protein modification
Protein or macromolecule localization
Chromosome organization
Protein, peptide, amide or nitrogen compound transport
Response to DNA damage
Others
0
5
10
−log10(p-value)
1* regulation of nucleobase-containing compound metabolic process
2* negative regulation of nucleobase-containing compound metabolic process
Cluster 3: genes with a core-less promoter
regulation of RNA metabolic process
3*regulation of nucleobase-containing compound metabolic...
negative regulation of RNA metabolic process
regulation of RNA biosynthetic process
transcription, DNA-templated
regulation of transcription, DNA-templated
regulation of nucleic acid-templated transcription
regulation of transcription from RNA polymerase II promoter
nucleic acid-templated transcription
establishment of protein localization
establishment of localization in cell
cellular localization
intracellular transport
protein transport
peptide transport
nitrogen compound transport
vesicle coating
amide transport
vesicle targeting, to, from or within Golgi
COPII-coated vesicle budding
RNA or DNA metabolic process
Transcription
Regulation of transcription
Protein or macromolecule localization
Protein, peptide, amide or nitrogen compound transport
0
5
10
−log10(p-value)
3* regulation of nucleobase-containing compound metabolic process
Cluster 4: all genes
cellular component assembly
single-organism organelle organization
organelle assembly
regulation of cellular component organization
regulation of organelle organization
cell cycle
cell cycle process
embryo development
central nervous system development
embryo development ending in birth or egg hatching
cellular response to stress
cellular component biogenesis
organelle membrane fusion
regulation of cellular component biogenesis
vesicle fusion
Cellular component organization
Cell cycle
Development
Cellular response to stress or DNA damage
others
0
5
10
−log10(p-value)

Cluster 4: genes with a core-less promoter

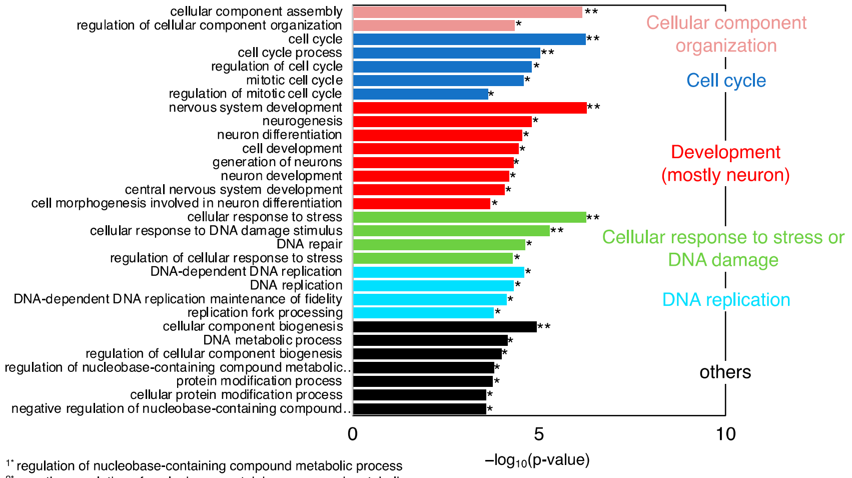

[1*] regulation of nucleobase-containing compound metabolic process
[2*] negative regulation of nucleobase-containing compound metabolic process

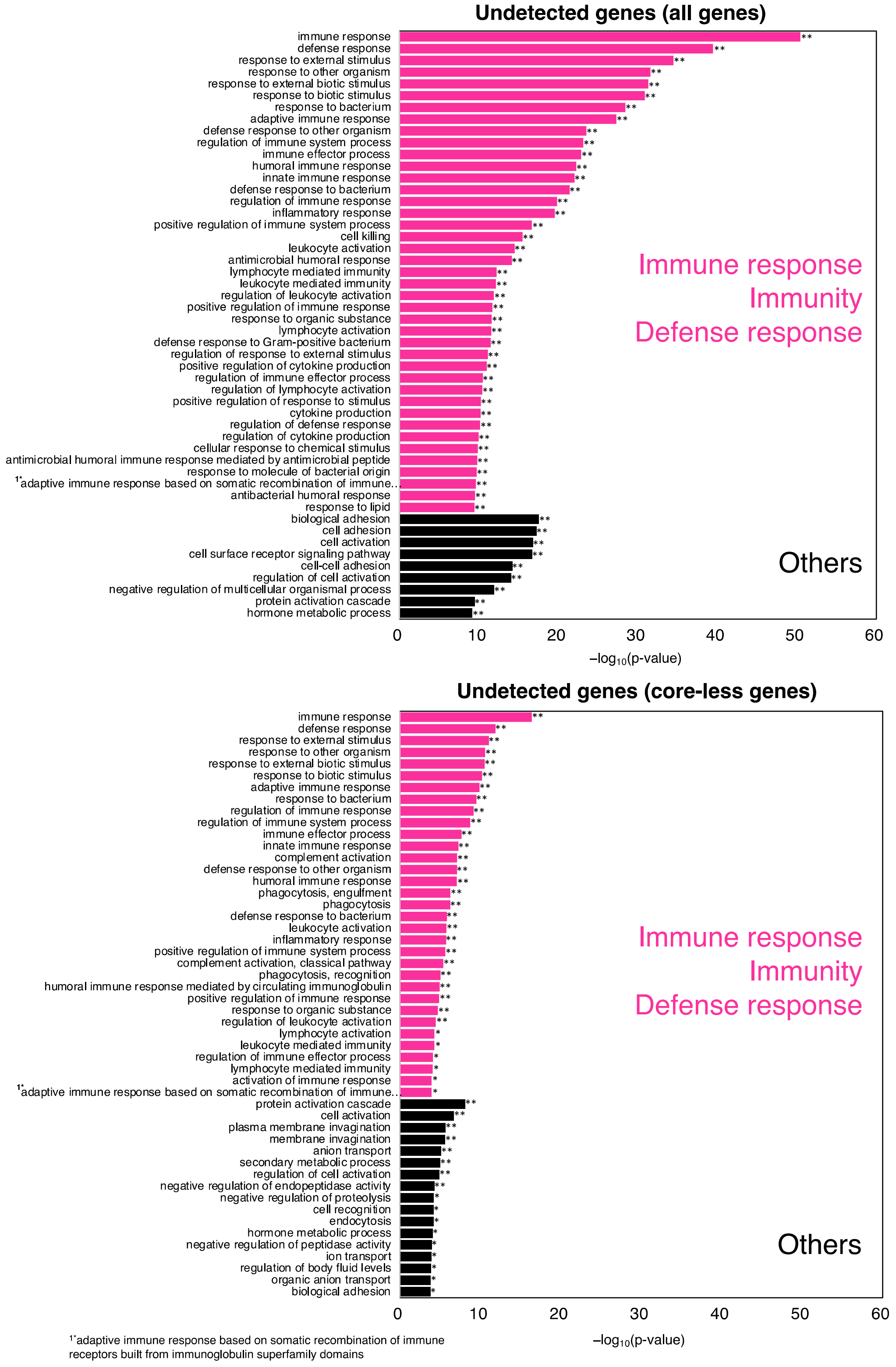

Supplementary Figure S2. Non-abbreviated version of Fig. 6a.
